# Azole-resistant *Aspergillus fumigatus* harboring TR_34_/L98H, TR_46_/Y121F/T289A and TR_53_ mutations related to flower fields in Colombia

**DOI:** 10.1038/srep45631

**Published:** 2017-03-30

**Authors:** Carlos Alvarez-Moreno, Rose-Anne Lavergne, Ferry Hagen, Florent Morio, Jacques F. Meis, Patrice Le Pape

**Affiliations:** 1 Departamento de Medicina Interna, Facultad de Medicina, Universidad Nacional de Colombia, Bogotá, Colombia; 2 Departamento Enfermedades Infecciosas, Clínica Universitaria Colombia Bogotá, Colombia; 3 Département de Parasitologie et Mycologie Médicale, Université de Nantes, Nantes Atlantique Universités, EA1155-IICiMed, Faculté de Pharmacie, Nantes, France; 4 Laboratoire de Parasitologie-Mycologie, Institut de Biologie, CHU de Nantes, France; 5 Department of Medical Microbiology and Infectious Diseases, Canisius Wilhelmina Hospital (CWZ), Nijmegen, The Netherlands; 6 Centre of Expertise in Mycology Radboudumc/CWZ, Nijmegen, The Netherlands

## Abstract

Resistance to triazoles in *Aspergillus fumigatus* has been reported in azole-naive patients in Europe, Asia, Australia and North America. This resistance has been linked to fungicide-driven mutations in the *cyp51A* gene and its promoter region. We investigated the presence of environmental azole-resistant *A. fumigatus* strains related to the use of azole fungicides in Colombia. Soil samples were collected from flower beds, flower fields and public gardens from the outskirts, suburbs and city centre of Bogotá. Out of the 86 soil samples taken, 17 (19.8%) grew *A. fumigatus* of whom eight (9.3%) contained 40 strains able to grow on azole-containing itraconazole and/or voriconazole supplemented media. All but one triazole-resistant strains were isolated from soil samples collected from flower fields and flower beds (39/40). Importantly, the majority had the TR_46_/Y121F/T289A, TR_34_/L98H, and TR_53_ molecular resistance mechanisms and one azole resistant strain had a wild-type *cyp51A* gene. Soil samples from flower fields and beds contained 4 azole fungicides (penconazole, difenoconazole, tetraconazole and tebuconazole) above the limit of detection. Our findings underline the need for extensive investigations to determine azole-resistant *A. fumigatus* prevalence in both clinical and environmental samples in other regions of Latin America.

Triazole-resistant *Aspergillus fumigatus* is increasingly reported in Europe since the late 2000s. This emerging public health concern occurs through two distinct pathways: *in vivo* selection of resistance as a consequence of long-term treatment with medical azoles or *de novo* acquisition of a resistant strain directly from the environment, linked to the widespread use of azole fungicides in agriculture. This second route of resistance acquisition was initially described in The Netherlands and then reported in several countries in Europe, Asia and Africa^1^,^2^,^3^ explaining the recent emergence of resistant *A. fumigatus* in azole-naive patients. In Europe, a relationship was found between environmental azole use and development of cross-resistance to medical triazoles through TR_34_/L98H and TR_46_/Y121F/T289A mutations in the *cyp51A* gene and its promoter region^4^. Recently, both the TR_34_/L98H and TR_46_/Y121F/T289A mutations were reported in *A. fumigatus* strains collected from several institutions throughout the United States^5^,^6^.

A large number of demethylation inhibitor (DMI) fungicides have been used intensively in agriculture since the 1970s^7^. More than thirty molecules are registered for each application field, but only a few are intrinsically active against *A. fumigatus*^7^,^8^. According to the Food and Agriculture Organization of the United Nations, Colombia was the fourth largest consumer of pesticides in arable lands and permanent crops in 2010 (14.5 tons per 1000 ha)^9^. Also, according to the pesticide report of the Ministry of Agriculture and Integrated Rural Development of Colombia for the period 2006–2013, fungicides ranked second in sales after herbicides (43 and 34%, respectively)^10^. Fungicides are authorized for use in a large variety of agricultural applications including flower production^11^.

Given this possible association between the use of DMI in agriculture and the emergence of azole-resistant *A. fumigatus*^3^,^4^,^12^,^13^ and the fact that Colombia is a country with a high flower production (second producer worldwide), we recently evaluated the presence of environmental resistance to azoles of medical importance in *A. fumigatus* isolated from flower fields and described the first environmental azole-resistant *A. fumigatus* strains in South America^14^. To gain further insights into this potential public health issue, we decided to conduct a complementary study by extending the sampling and to explore the genetic relationship between strains found in flower fields, ornamental plant beds and urban public gardens. In addition, concentrations of triazole fungicides used in Colombian floriculture were measured in the same soil samples. Finally, antifungal activity of flutriafol against wild-type *A. fumigatus* was also investigated.

## Results

**Figure 1:**
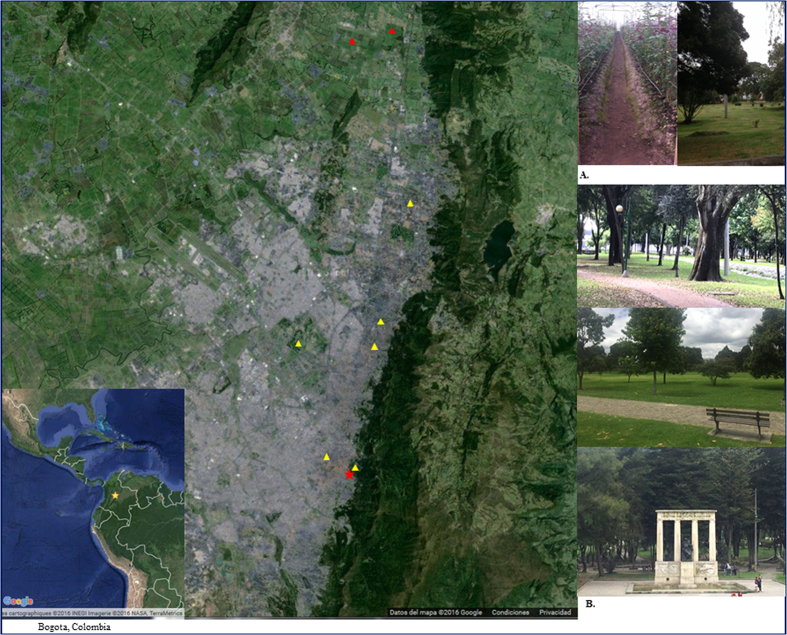
Localization of samplings: (**A**) outskirts, suburbs (flower fields and green houses, red triangles), which were positive to azole resistant *Aspergillus fumigatus*. (**B**) West, North and downtown areas of the city of Bogotá (public gardens, yellow triangles), which were negative, except National Park (red asterisk). Map data: Google GeoBasis-DE/BKG and Google, INEGI.

After analysis of the fungicides applied in Colombia, as well as their use in agriculture, the horticulture sector was selected because of its great number of combined or single fungicide molecules approved (about thirty distinct compounds in 2015). Soil samples (*n* = 86) from nine different flower fields (*n* = 32) and flower beds (*n* = 12) in the outskirts of Bogotá and public gardens (*n* = 42) of the city centre were collected (Fig. 1). From the 86 soil samples, 17 (19.8%) were positive for *A. fumigatus* on Sabouraud Dextrose Agar plates among of which 8 exhibited strains able to grow on supplemented agar with itraconazole or voriconazole (*n* = 40 single colonies). Most triazole-resistant strains were detected in soil samples from flower fields and flower beds (*n* = 39/40) whereas only one strain was isolated from one of the inner city soil samples (Fig. 1). Of the 40 azole-resistant *Aspergillus* section *Fumigati* strains, 21 (up to 5 colonies for each positive culture) were identified as *A. fumigatus* by sequencing of the β-tubulin gene and further selected to analyze resistance mechanisms. Results showed a great diversity in molecular resistance mechanism with a larger number of strains harbouring TR_46_/Y121F/T289A (*n* = 17; 80.9%) followed by TR_53_ (*n* = 2) and TR_34_/L98H (*n* = 1) and finally one azole resistant strain was *cyp51A* wild-type. All *A. fumigatus* strains harbouring the TR_46_/Y121F/T289A mutation grew on voriconazole and itraconazole plates whereas TR_53_, TR_34_/L98H and the *cyp51A* wild-type strains grew only on itraconazole-containing agar plates.

**Figure 2:**
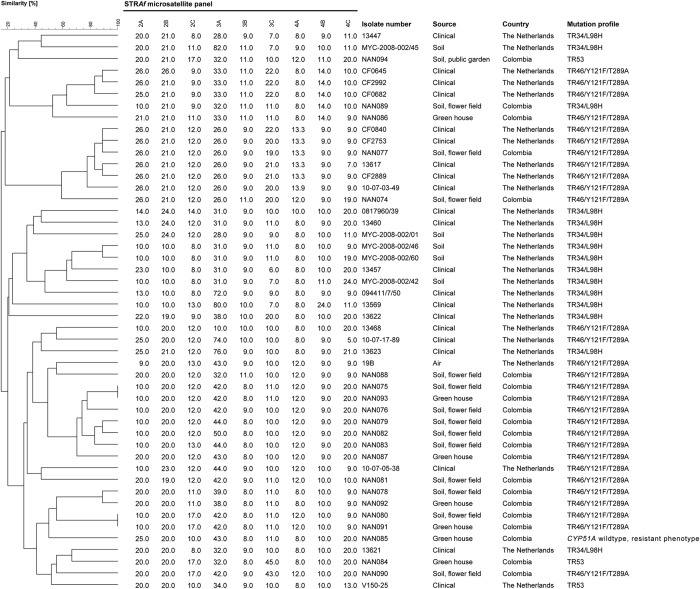
Genotypic relationship of *A. fumigatus* strains from environmental samples collected from the surroundings of a outskirts, suburbs and downtown areas of the city of Bogotá, Colombia (including TR_46_/Y121F/T289A, *n* = 17; TR_34_/L98H, *n* = 1;TR_53_, *n* = 2 and wild-type, *n* = 1) with resistant strains from the Netherlands (TR_46_/Y121F/T289A, 5 clinical and 1 environmental strains; TR_34_/L98H, 9 clinical and 5 environmental strains and one TR_53_ clinical strain). The dendrogram is based on a categorical analysis of nine microsatellite markers in combination with UPGMA clustering.

Strikingly, microsatellite genotyping of the azole-resistant strains from Colombia highlighted a large genotypic diversity, not only among strains with different molecular resistance mechanisms (*cyp51A* wild type, TR_34_/L98H and TR_53_) but also in those with TR_46_/Y121F/T289A (Fig. 2). The Colombian TR_46_/Y121F/T289A strains were genetically unrelated to Dutch TR_46_/Y121F/T289A clinical and environmental strains as was the single Colombian TR_34_/L98H strain. Notably, some isolates taken in flower fields were genetically undistinguishable from others collected in flower beds in the outskirts of Bogotá, located a few kilometres apart. The rarely occurring TR_53_ resistance mechanism was found in only two strains (flower field soil and public garden soil) in the city centre. Genotyping showed these strains to be different from each other and from the single, previously published, Dutch strain.

**Figure 3:**
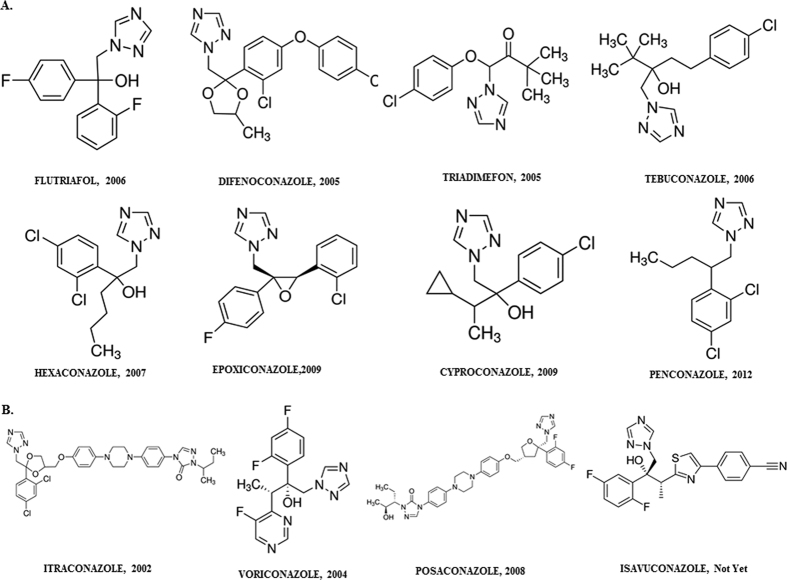
Structures of triazoles used in Colombian floriculture (**A**). Registration date of authorization for use in floriculture is provided after the name of the active product. (**B**) Clinical azole antifungal drugs with activity against aspergilli and to the right of the name the dates of clinical use authorization in Colombia. Source: National Center for Biotechnology Information. PubChem Compound Database; URL: https://pubchem.ncbi.nlm.nih.govCID=86132, https://pubchem.ncbi.nlm.nih.gov/compound/86132 (accessed Apr. 16, 2016).

Azole fungicides currently approved for use in Colombia in floriculture and azole antifungal drugs with activity against aspergilli were reported according to the date of registration for floriculture (Fig. 3). Because of the lack of data on antifungal activity of flutriafol against *A. fumigatus*, this DMI and tebuconazole (comparator) MICs were evaluated against 16 wild-type strains. Flutriafol had no activity against any of the *A. fumigatus* tested (MICs > 8 mg/L) while tebuconazole MICs ranged from 2 to 8 mg/L.

Finally, azole fungicides were measured in two representative pooled soil samples with LC-MS/MS. The results showed that in the soil of flower crops, the presence of penconazole (0.15 mg/kg), difenoconazole (0.41 mg/kg), tetraconazole (0.49 mg/kg) and tebuconazole (0.69 mg/kg) were found while the public garden soils had no detectable presence of any fungicide.

## Discussion

Fungal plant diseases cause extensive losses of crops in every area of human agriculture. Currently close to 150 different fungicidal compounds, formulated and sold in a several fold larger number of different proprietary products, are used worldwide in agriculture^8^,^12^. In Colombia, many fungicides are currently licensed for crop treatment with a number of chemical groups available in floriculture such as acylanines, methocy-acylates, carboxamides, carbamates, phthalimides, imidazoles, triazoles, to be used against *Botrytis cinerea* (chrysanthemum, carnation and rose crops), *Cladosporium echinolatum* and *Uromyces dianthi* (Carnation), *Sphaerotheca pannesa, Phramidium mucronatum* (rose), *Puccinia chrysanthemi* (chrysanthemum), among others. Triazole fungicide consumption remains high in Colombia, although there is a decrease in recent years. In the year 2000 fungicides accounted for 36% of total pesticide sales, of which 80% were triazoles. During the period 2006–2013, fungicides constituted 34% of pesticide sales with 23.7 and 36% in amount of number of litres and tons, respectively^10^. It is emphasized that triazoles such as bromuconazole, difenoconazole, epoxiconazole, propiconazole and tebuconazole in 2003 were 20% of total consumption of fungicides in Colombia compared to 15.8% in 2013 (7.2 and 8.6% as simple or combined products)^10^,^11^,^15^. Interestingly among the eight triazoles registered for use in floriculture in Colombia, flutriafol (this study), cyproconazole, penconazole and triadimefon have no activity against *A. fumigatus* and of the four compounds remaining, only hexaconazole has limited activity. The remaining three (difenoconazole, tebuconazole, epoxiconazole) with chemical similarity to the medical triazoles exhibited high activity against *A. fumigatus*. They have already been listed as possible candidates to induce resistance in *Aspergillus*, especially tebuconazole^4^,^16^,^17^. The soil samples analysis showed the presence of 3 triazole fungicides (tebuconazole, difenoconazole and tetraconazole). It is noteworthy that the latter is not registered for use in the flower industry^11^. Although *in vitro* induction of alterations in the promoter region following *in vitro* exposure to DMIs was not successful^4^, the fact that we found azole-resistant *A. fumigatus* in the same soil samples increases suspicion of an association among these fungicides and the spread of *Aspergillus* resistance in floriculture.

Notably, our study on azole resistance in *A. fumigatus* showed a great diversity of resistance mechanisms with a predominance of TR_46_/Y121F/T289A over TR_34_/L98H and TR_53_. All three resistance mechanisms were also genotypically different from strains with the same mutations from the Netherlands. This study is the first to demonstrate the highest frequency of this mutation in environmental strains in contrast to the formerly reported in Europe, Asia and Africa^2^,^3^,^18^,^19^. This work is also the first report of its association with soil of flower fields in South America. Since Colombia is the second exporter of cut flowers, we also analyzed whether dissemination could be possible from cut flowers but did not find any *A. fumigatus* on selected samples (unpublished data).

Although these mutations target an important biological function in the fungus, fitness cost has not been demonstrated for TR_34_/L98H and TR_46_/Y121F/T289A. Moreover, the presence of two or three genetic alterations respectively could stabilize enzyme function compared with only one or two of these mutations^20^,^21^. Under selection pressure and prolonged exposure to fungicides, mutant strains could have greater resilience and may even predominate in the environment^17^. Finally, the impact of the TR_53_ mutation which has been found only once in clinical samples^22^, and once in the environment in Netherlands^23^ (data not published) has not yet been established, but considering its low frequency could have limitations on fungal adaptation to the environment. Although we only found two strains with this mutation not related to each other, in Colombia it could have achieved its adaptation to the environment.

The selection of TR_46_/Y121F/T289A alteration during long-term voriconazole therapy has been suggested recently^24^. However, in the absence of convincing proof it is now well accepted that these alterations are environmental DMI fungicide-driven^25^. It is worth noting that a risk assessment of environmental resistance selection to DMIs during exposure in floriculture has been considered as a medium risk compared to veterinary and human use but a major risk in comparison to other agricultural practices^7^. Nevertheless, this exposure depends in part on the half-life of a fungicide that is conditioned by a number of factors including climate, soil composition, nature of the chemical, and number of fungicide applications. Because there are no specific growth seasons in tropical countries such as Colombia, the frequency of fungicide applications seems to be higher and could partly reflect the high consumption in this country. These frequent applications and the fact that triazole fungicides can persist a long time in soil^26^,^27^ could explain resistance development and the diversity of resistance mechanisms. Furthermore, it is unclear whether in real life conditions, fungicides utilized out of the established indications (different application frequency, too low doses, inappropriately used compounds according to the crops, etc.) could have facilitated TR_46_/Y121F/T289A predominance. Some limitations of this study reside in the lack of data regarding DMIs used in the studied flower fields and flowers beds, the frequency of use, the application procedures (bulb dipping, spraying or both) and possible rotations. Soil fungicide quantification was done in two representative pooled samples.

Our finding that two distinct sampling sites exhibited genotypically undistinguishable strains could be explained by clonal spread. Because resistance could also be induced in other crops in the same region where DMIs are used, we are currently conducting a study aimed at evaluating the presence of azole-resistant *A. fumigatus* related to potatoes, corn, carrots, beans, and other vegetables.

According to the Fungicide Resistance Action Committee (FRAC), among the possible mechanisms of generation of resistance in plant pathogenic fungi, all the models indicate that use of both fungicide mixtures and rotations can delay, but not prevent, emergence of resistant mutants^8^. It is also noted that in recent years fungicide consumption has decreased in Colombia but, and in parallel, has seen an increase in the consumption of fungicide combinations^11^,^15^. It should be noted that with the exception of epoxiconazole (2009) and flutriafol (2006), all other fungicides have been marketed in Colombia since the early nineties while the medical azole antifungal drugs (itraconazole, voriconazole, posaconazole) were introduced to clinical use in (2000, 2004 and 2008 respectively) the first decade of the 21st century (Fig. 3).

We also found a *cyp51A* wild-type strain with an azole-resistant phenotype, which is not surprising considering that multiple mechanisms of resistance have already been described such as increased drug efflux and a *HapE* mutation^28^,^29^.

Because prevalence of azole-resistant *A. fumigatus* is increasing around the world^2^,^3^,^13^, despite it has not been possible to demonstrate specifically the relationship between some fungicide triazoles and the emergence of resistance, is important to establish azole resistant control policies in different regions of the world. For the World Health Organization, it is a priority to delay antimicrobial resistance development and although current strategies are still focused on antibiotics, many of which could have a similar impact, not only on azole compounds used in agriculture, but also in human and animal health. It is likely that with the current information only restricting the use or stimulating the adherence to technical recommendations and establishing active surveillance of one or two fungicides and not a whole family would be enough. Indeed, this strategy has proved successful in the control of Extended-Spectrum Beta-Lactamases (ESBLs) in Enterobacteriaceae through the restriction of ceftazidime in favour of other beta-lactams instead^30^,^31^. Finally, restrictions of fungicides at an appropriate time could have the desired effect on preserving human antifungal therapy options in the future. In response to the emergence of human vancomycin resistant *Enterococcus faecium* (VRE), avoparcin, a vancomycin analogue used as an animal growth enhancer was discontinued leading to a decrease in the prevalence of VRE among farm animals^32^.

Albeit the study findings cannot be generalized to all the Colombian floriculture and much less to the overall situation of the country, they suggest that azole-resistant *A. fumigatus* is at the present time becoming a potential public health issue. Indeed, until recently, the only region in the world which had not reported the presence of resistance was Latin America. Now it is necessary to evaluate the true prevalence in this region and what the real impact is on human health. In this regard, it is important to realize that all TR_46_/Y121F/T289A strains are resistant to voriconazole, the first-line treatment for patients with invasive aspergillosis^16^,^18^,^33^. In addition, azole resistant *A. fumigatus* harbouring TR_46_/Y121F/T289A are also resistant to isavuconazole, the new medical antifungal compound which is considered a good therapeutic option for aspergillosis^34^. Currently we are evaluating the incidence of *A. fumigatus* on exacerbations during chronic obstructive pulmonary disease in the same region.

In conclusion, our study showed evidence of a larger geographical spread of azole resistance in *A. fumigatus* now also in Latin America. These findings also highlight the great diversity in molecular resistance mechanisms and the predominance of TR_46_/Y121F/T289A strains associated with high-level resistance to voriconazole, a first-line treatment for patients with invasive aspergillosis. Finally, these data underline the need for targeted investigations to determine the prevalence of azole resistance in *A. fumigatus* in both clinical and environmental samples in other regions of Colombia and Latin America.

## Methods

### Study Design

To recover *A. fumigatus* strains from the outdoor environment, 2 g of each soil sample was suspended in 0.85% NaCl plus 0.01% Tween20, vortexed and allowed to settle, as described previously^4^. From the supernatant, 50 μl was plated on Sabouraud dextrose agar (SDA) and incubated at 43 °C for 48 hours to select *Aspergillus fumigatus. Aspergillus* section *Fumigati* identification was obtained by both macroscopic and microscopic characteristics.

### Azole Resistance Detection

To detect azole-resistant *A. fumigatus*, conidia were suspended in 0.85% NaCl plus 0.01% Tween20 at a 0.5 McFarland turbidity and 50 μl of inoculum suspensions were added on Sabouraud agar plates containing 4 mg/L of either itraconazole or voriconazole (both Sigma–Aldrich, Saint-Quentin Fallavier, France) and they were then incubated at 37 °C for 48 h^35^. Up to 5 colonies for each positive culture were selected and genomic DNA was extracted from the azole-resistant strains using the NucleoSpin^®^ Microbial DNA procedure (Macherey-Nagel GmbH & Co., Düren, Germany) according to the manufacturer’s instructions.

### Mutation Analysis

Azole-resistant *A. fumigatus sensu stricto* strains were identified by amplification and sequencing of a portion of the β-tubulin gene^36^. To study the resistance mechanism involved in these strains, the *cyp51A* gene and a 234 bp region of its promoter were further analyzed by sequencing as described previously^37^. Nucleotide sequences were compared with the reference sequence of the *A. fumigatus* azole-susceptible strain CM-237 (GenBank accession number AF338659) using Seqscape (Applied Biosystems, Foster City, CA) for *cyp51A* gene and ClustalW analysis by means of BioEdit^®^ software version 7.1.11.0 (Carlsbad, CA) for the promoter region.

### Microsatellite Typing

Genotyping was performed on all *A. fumigatus* strains able to grow on 4 mg/L itraconazole or voriconazole, using microsatellite typing, as described previously^38^. The fragment sizes were determined by addition of the CC-ROX500 marker and subsequent analysis of the fragments on the Applied Biosystems ABI3500xL Genetic Analyzer. Assignment of repeat numbers for each of the nine markers was determined by using the GeneMapper package version 1.0 software (Applied Biosystems). Repeat numbers of these strains were compared to a collection of Dutch TR_46_/Y121F/T289A (*n* = 12), TR_34_/L98H (*n* = 14) and TR_53_ (*n* = 1) positive strains. Allele sharing distance matrices were generated from the tandem repeat numbers and were used as input to the Neighbor program of the PHYLIP version 3.6 software package to produce dendrograms.

### Fungicide detection

Soil samples were examined for the following azole fungicides: azaconazole, cyproconazole, difenoconazole, epoxyconazole, fluquinconazole, flusilazole, flutriafol, hexaconazole, penconazole, propiconazole, tebuconazole, tetraconazole and triadimenole using liquid chromatography-tandem mass spectrometry (LC-MS/MS) with a limit of quantification (LOQ) of 0.05 mg/kg^39^. Briefly, dried, finely homogenized soil samples (10 g) were extracted by the Dionex ASE 200 system (Thermo Fisher Scientific, France) using methanol/dichloromethane (85:15, v/v) as solvent. Extracts were evaporated under a stream of nitrogen before the solid phase extraction (SPE) using Oasis^®^ MAX (6 mL, 150 mg) from Waters (Guyancourt, France). The cartridges were conditioned with 6 mL dichloromethane and then 6 mL of methanol and the elution was performed with two volumes of 5 mL, firstly 100% methanol and then dichloromethane. After evaporation under a stream of nitrogen, internal standard fungicide was added and the mixture evaporated to dryness. Following the analytical protocol, the sample extract was reconstituted in 500 μl of water:acetonitrile (90:10, v/v). High performance liquid chromatography (HPLC) was performed with a Gemini-NX C18 3 μm, 100 mm × 2 mm with a SecurityGuard cartridge Gemini-NX C18 3 μm, 4 mm × 2.0 mm, both from Phenomenex (Le Pecq, France). Acetonitrile and 5 mM ammonium acetate solution were used with an analytical gradient of 15 min. The detector was a API 2000 triple quadrupole (Applied Biosystems/MDS/SCIEX, Villebon sur Yvette, France) mass spectrometer equipped with an electrospray ionization source (ESI) that was operated in the positive ionization mode. Mass acquisition was performed in the sSRM mode. Analyst software 1.5.2 was used with the SRM algorithm for acquiring and interpreting the results.

### Antifungal Susceptibility Testing

Antifungal activity (MIC) of flutriafol used in Colombian floriculture was investigated against wild-type and azole-resistant strains using the European Committee for Antimicrobial Susceptibility Testing (EUCAST) standard method for moulds^40^. Tebuconazole was used as comparator.

## Additional Information

**How to cite this article:** Alvarez-Moreno, C. *et al*. Azole-resistant *Aspergillus fumigatus* harboring TR_34_/L98H, TR_46_/Y121F/T289A and TR_53_ mutations related to flower fields in Colombia. *Sci. Rep.* **7**, 45631; doi: 10.1038/srep45631 (2017).

**Publisher's note:** Springer Nature remains neutral with regard to jurisdictional claims in published maps and institutional affiliations.
